# Synaptic Terminal Density Early in the Course of Schizophrenia: An In Vivo UCB-J Positron Emission Tomographic Imaging Study of SV2A

**DOI:** 10.1016/j.biopsych.2023.05.022

**Published:** 2024-04-01

**Authors:** Ellis Chika Onwordi, Thomas Whitehurst, Ekaterina Shatalina, Ayla Mansur, Atheeshaan Arumuham, Martin Osugo, Tiago Reis Marques, Sameer Jauhar, Susham Gupta, Ravi Mehrotra, Eugenii A. Rabiner, Roger N. Gunn, Sridhar Natesan, Oliver D. Howes

**Affiliations:** a Institute of Clinical Sciences, Faculty of Medicine, Imperial College London, London, United Kingdom; b Psychiatric Imaging Group, Medical Research Council, London Institute of Medical Sciences, Hammersmith Hospital, London, United Kingdom; c Department of Psychosis Studies, Institute of Psychiatry, Psychology & Neuroscience, King’s College London, London, United Kingdom; d Centre for Psychiatry and Mental Health, Wolfson Institute of Population Health, Queen Mary University of London, London, United Kingdom; e Department of Brain Sciences, Imperial College London, The Commonwealth Building, Hammersmith Hospital, London, United Kingdom; f Invicro, Burlington Danes Building, London, United Kingdom; g Department of Psychological Medicine, Institute of Psychiatry, Psychology & Neuroscience, King’s College London, London, United Kingdom; h Early Detection and Early Intervention, East London National Health Service Foundation Trust, London, United Kingdom; i Early Intervention in Psychosis Team, West Middlesex University Hospital, West London National Health Service Trust, Isleworth, London, United Kingdom; j Centre for Neuroimaging Sciences, Institute of Psychiatry, Psychology & Neuroscience, King’s College London, London, United Kingdom

**Keywords:** Antipsychotic-free, Positron emission tomography, Schizophrenia, SV2A, Synaptic density, UCB-J

## Abstract

**Background:**

The synaptic hypothesis is an influential theory of the pathoetiology of schizophrenia (SCZ), which is supported by the finding that there is lower uptake of the synaptic terminal density marker [^11^C]UCB-J in patients with chronic SCZ than in control participants. However, it is unclear whether these differences are present early in the illness. To address this, we investigated [^11^C]UCB-J volume of distribution (*V*_T_) in antipsychotic-naïve/free patients with SCZ who were recruited from first-episode services compared with healthy volunteers.

**Methods:**

Forty-two volunteers (SCZ *n* = 21, healthy volunteers *n* = 21) underwent [^11^C]UCB-J positron emission tomography to index [^11^C]UCB-J *V*_T_ and distribution volume ratio in the anterior cingulate, frontal, and dorsolateral prefrontal cortices; the temporal, parietal and occipital lobes; and the hippocampus, thalamus, and amygdala. Symptom severity was assessed in the SCZ group using the Positive and Negative Syndrome Scale.

**Results:**

We found no significant effects of group on [^11^C]UCB-J *V*_T_ or distribution volume ratio in most regions of interest (effect sizes from *d* = 0.0–0.7, *p* > .05), with two exceptions: we found lower distribution volume ratio in the temporal lobe (*d* = 0.7, uncorrected *p* < .05) and lower *V*_T_/*f*_p_ in the anterior cingulate cortex in patients (*d* = 0.7, uncorrected *p* < .05). The Positive and Negative Syndrome Scale total score was negatively associated with [^11^C]UCB-J *V*_T_ in the hippocampus in the SCZ group (*r* = −0.48, *p* = .03).

**Conclusions:**

These findings indicate that large differences in synaptic terminal density are not present early in SCZ, although there may be more subtle effects. When taken together with previous evidence of lower [^11^C]UCB-J *V*_T_ in patients with chronic illness, this may indicate synaptic density changes during the course of SCZ.

SEE COMMENTARY ON PAGE 605

Multiple lines of evidence suggest that synaptic dysfunction plays a central role in schizophrenia (SCZ) pathogenesis. Initial reports concerned postmortem evidence of lower dendritic spine density (1,2), lower synaptic protein and messenger RNA levels (3), and genetic evidence (4, 5, 6). Recently, our group reported in vivo evidence for lower synaptic terminal density indexed using [^11^C]UCB-J imaging in patients with chronic SCZ than in healthy control participants (7). These findings were subsequently replicated by a separate group (8).

However, because the [^11^C]UCB-J imaging studies were conducted in patients with chronic SCZ, it is unknown whether synaptic deficits emerge early in the course of illness or develop later during the illness.

Therefore, we conducted a clinical imaging study using [^11^C]UCB-J positron emission tomography (PET) to test the hypothesis that [^11^C]UCB-J volumes of distribution (*V*_T_) would be lower in patients with early-course SCZ than in control participants in brain regions where evidence for lower synaptic terminal density has been reported in patients with chronic SCZ (3,7,8). We also tested the hypothesis that [^11^C]UCB-J *V*_T_ would be inversely associated with symptom severity.

## Methods and Materials

The London-West London & GTAC Research Ethics Committee, United Kingdom (reference: 16/LO/1941) approved the study protocol. The Administration of Radioactive Substances Advisory Committee, United Kingdom, approved the administration of radioactive material. We obtained written informed consent from all volunteers before their participation in the study, which was conducted in accordance with the Declaration of Helsinki (1996).

We recruited 42 volunteers (21 healthy volunteers [HVs] through public advertisement and 21 patients). PET and clinicodemographic data for 17 healthy volunteers [in analyses in (7,9)], but none of the SCZ group, have been reported previously. Inclusion criteria for all volunteers were 18 to 65 years of age, capacity to consent, and a normal blood coagulation test.

Patients were recruited from London first-episode psychosis services. Inclusion criteria were meeting DSM-5 criteria for SCZ and being antipsychotic-naïve or free from antipsychotic medication for at least 4 weeks before [^11^C]UCB-J imaging. HVs had to have no history of a mental disorder or family history of SCZ (see Supplemental Methods for exclusion criteria).

### Clinical Assessments

The Structured Clinical Interview for DSM-5 and the Positive and Negative Syndrome Scale (PANSS) (10) were administered. Illness duration was determined as the time from each patient’s first psychotic symptoms. See Supplemental Methods for additional information.

### Magnetic Resonance Imaging

All subjects underwent structural magnetic resonance imaging (MRI) to facilitate the anatomical delineation of regions of interest (ROIs) (see Supplemental Methods).

### PET Acquisition and Analysis

#### PET Imaging

Following a low-dose computed tomography scan for attenuation and scatter correction, subjects received a microdose of [^11^C]UCB-J (≤300 MBq) as a smooth bolus injection via an intravenous cannula for 20 seconds. A Biograph 6 HiRez PET-CT scanner (Siemens) was used to acquire PET data for 90 minutes.

#### Arterial Blood Sampling

Radial arterial blood samples were collected throughout the PET scan to measure the arterial input function as has been detailed elsewhere (11). Briefly, a continuous automatic blood sampling system was used to measure whole-blood activity for the first 15 minutes (Allogg AB) (see Supplemental Methods for details).

#### Image Analysis

Processing and modeling were conducted using MIAKAT version 4.3.7 (http://www.miakat.org/MIAKAT2/index.html), which was implemented in MATLAB (version R2018b; The MathWorks, Inc.) with functions from FSL (version 5.0.10; FMRIB) and SPM12 (Wellcome Trust Centre for Neuroimaging, http://www.fil.ion.ucl.ac.uk/spm).

Each subject’s MRI underwent brain extraction using FSL and gray matter segmentation and rigid-body coregistration to a standard reference space (12) using SPM12 as implemented via MIAKAT. The template brain image and associated Clinical Imaging Centre atlas (13) were then warped nonlinearly to the subject’s MRI. The frontal cortex (FC), anterior cingulate cortex (ACC), and hippocampus were defined as primary ROIs based on previous imaging and postmortem evidence for lower synaptic protein levels in these regions (3,7, 8, 9). We used the same atlas to define the occipital, parietal, and temporal lobes, dorsolateral prefrontal cortex, thalamus, and amygdala as exploratory ROIs because they have been implicated in SCZ pathophysiology (7,8,14, 15, 16). The centrum semiovale (CS) ROI was generated from the automated anatomical labeling template (17) according to parameters defined for its use as a reference region to estimate nondisplaceable [^11^C]UCB-J binding (18).

PET images were registered to each subject’s MRI and motion corrected through frame-to-frame rigid-body registration using the 14th frame (acquired 9–11 minutes after injection) as the reference frame. The extent of motion was assessed during the scan, and registration parameter plots were derived. Total movement was defined as total frame-by-frame Euclidean distance, which was estimated from frame realignment during motion correction. Time activity curves were generated for each ROI.

Regional time activity curves and arterial input function data were analyzed together using the 1-tissue compartment model, which produces reliable [^11^C]UCB-J *V*_T_ estimates (11,19).

Gray matter masks were applied to ROIs within MIAKAT to extract regional gray matter *V*_T_. Regional distribution volume ratio (DVR) was obtained using the CS as a pseudoreference region (11,18), thus deriving DVR as a ratio of ROI *V*_T_ to CS *V*_T_.

### Sample Size and Power Calculation

We calculated the minimum sample size needed to test our primary hypothesis using G∗power version 3.1.9.3 (https://www.psychologie.hhu.de/arbeitsgruppen/allgemeine-psychologie-und-arbeitspsychologie/gpower). Previous studies identified lower [^11^C]UCB-J binding in patients with SCZ than in control participants, with effect sizes around 0.9 or greater in our primary ROIs (7,8). The power calculation indicated that a minimum sample size of 21 subjects per group would have more than 80% power to detect a significant group difference in [^11^C]UCB-J binding with Cohen’s *d* ≥ 0.9.

### Statistical Analysis

Statistical analyses were conducted using GraphPad Prism version 8.00 for Mac (GraphPad Software, http://www.graphpad.com), IBM SPSS Statistics version 25 and RStudio Version 1.1.456 (RStudio Team [2016], RStudio, Inc., http://www.rstudio.com/). We tested for normality of distribution using the Shapiro-Wilk test. When data were normally distributed, we used two-way repeated measures analysis of variance to test group and ROI effects. We used planned independent sample *t* tests (two-tailed) to test the effect of group on *V*_T_ at each ROI, applying a false discovery rate (FDR) correction with Q = 5% to limit false discoveries when performing multiple group comparisons (20). When data were not normally distributed, we used nonparametric analyses to test for group effects. We assessed group differences in clinicodemographic variables using independent sample *t* tests, χ^2^ tests, and Kolmogorov-Smirnov tests for normally distributed, categorical, and nonparametric data, respectively. We tested whether there were significant associations between *V*_T_ and PANSS total scores using Pearson’s product-moment correlation coefficients for normally distributed data and Spearman’s rank correlations for non-normally distributed data. When an association was significant, we conducted exploratory analyses to test whether there were associations between *V*_T_ and PANSS positive, negative, and general subscale scores.

For comparisons with previous studies, we conducted exploratory analyses using the same statistical approach to assess whether there were significant alterations in SCZ using [^11^C]UCB-J *V*_T_ in other ROIs and using [^11^C]UCB-J DVR as the outcome measure instead of *V*_T_.

Exploratory voxelwise whole-brain analyses were conducted using SPM12 to determine whether there were alterations in [^11^C]UCB-J *V*_T_ that were not detected by the ROI analysis (see Supplemental Methods).

## Results

Forty-two volunteers (*n* = 21 HVs and *n* = 21 with SCZ) completed the study. There were no significant group differences in age, sex, ethnicity, proportion of current smokers, or cannabis use during the past month or in [^11^C]UCB-J injected activity, injected cold mass, specific radioactivity, minimum purity, or plasma-free fraction (*f*_p_) (Table 1).

**Table 1:** Clinicodemographic and Imaging Variables in the HV and SCZ Groups

	HV	SCZ	Test Statistic	*p*
Age, Years	30.86 (1.90) [20–49]	26.52 (1.74) [18–48]	*z* = 1.08	.19
Sex, Female/Male	5/16	4/17	χ^2^_1_ = 0.14	.71
Ethnicity, Asian/Black/Other/White	3/5/1/12	4/11/2/4	χ^2^_3_ = 6.73	.08
Current Smoker	5	7	χ^2^_1_ = 0.47	.49
Cannabis Users Within the Last Month	1	2	χ^2^_1_ = 0.36	.55
Activity Injected, MBq	262.96 (4.94) [213.47–295.90]	221.24 (12.84) [107.20–280.46]	*z* = 1.23	.10
Injected Mass, μg	3.11 (0.22) [1.62–4.67]	3.18 (0.35) [1.12–8.27]	*z* = 0.46	.98
Specific Radioactivity, GBq/μM	30.41 (2.50) [15.27–51.84]	25.64 (2.21) [10.37–43.76]	*t*_40_ = 1.43	.16
Minimum Purity Fraction	99.97 (0.03) [99.34–100]	100 (0.00) [100–100]	*z* = 0.15	>.99
[^11^C]UCB-J Plasma-Free Fraction	0.24 (0.005) [0.20–0.29]	0.27 (0.007) [0.23–0.38]	*z* = 1.23	.10
Total Motion During PET Scan, mm	23.30 (5.87) [1.24–92.85]	22.50 (6.12) [0.60–120.57]	*z* = 0.77	.59
Illness Duration, Years	–	2.67 (0.46) [1–9]	–	–
Drug-Free Interval, Days	–	180.42 (27.71) [41–448]	–	–

Values are mean (SEM) [range] or *n*.

HV, healthy volunteer; PET, positron emission tomography; SCZ, schizophrenia.

None of the SCZ volunteers were taking antipsychotic medication. Two were antipsychotic-naïve, and 19 had taken antipsychotic medication previously, with a mean (SEM) interval of 180.42 (27.71) days and a minimum of 41 days between the most recent antipsychotic dose and [^11^C]UCB-J imaging. None of the SCZ volunteers had comorbid DSM-5 psychiatric diagnoses.

### [^11^C]UCB-J *V*_T_ Across Groups in Primary ROIs

**Figure 1:**
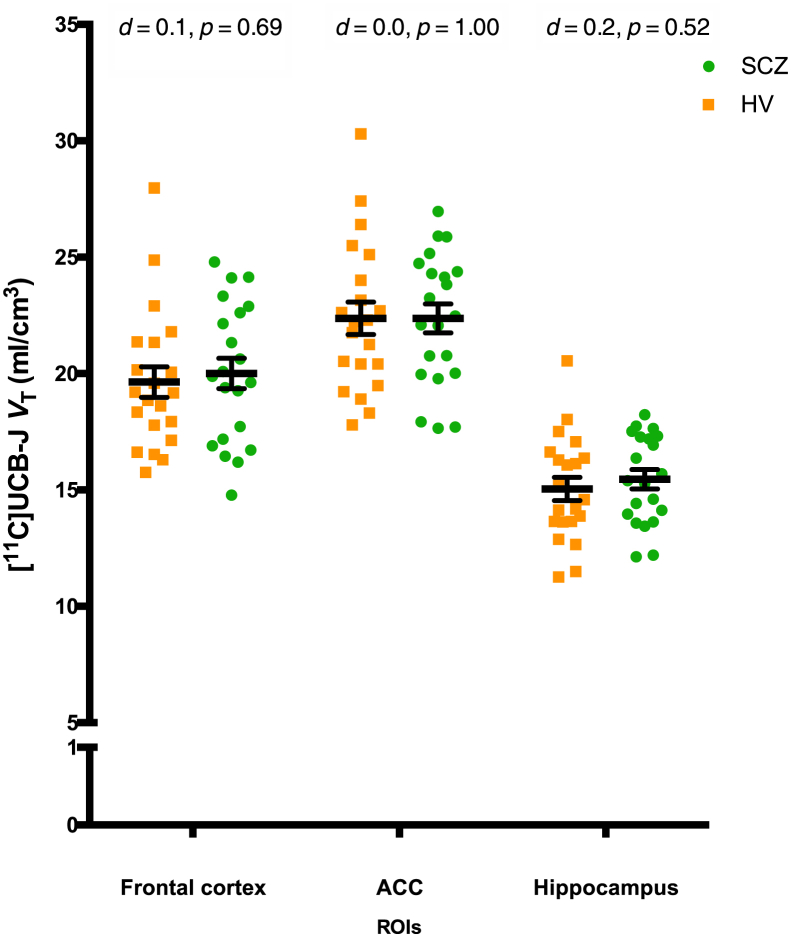
[^11^C]UCB-J volume of distribution (*V*_T_) in the frontal cortex, anterior cingulate cortex (ACC), and hippocampus by group. Orange squares indicate the healthy volunteer (HV) group (*n* = 21); green dots indicate the schizophrenia (SCZ) group (*n* = 21). [^11^C]UCB-J *V*_T_ was not significantly altered in the SCZ group compared with the HV group in any region of interest (ROI). Horizontal bar indicates mean; error bars indicate standard error of the mean.

Data were normally distributed in each ROI in each group. There was a significant effect of ROI (*F*_2,40_ = 609.4, *p* < .0001) but not of group (*F*_1,20_ = 0.09, *p* = .77; even with age as a covariate [*F*_1,39_ = 0.68, *p* = .41]) on [^11^C]UCB-J *V*_T_ (Figure 1). Post hoc analyses with FDR adjustment revealed no significant group differences in any ROI (Figure 1; Table S1).

### Relationship Between [^11^C]UCB-J *V*_T_ and Symptom Severity

**Figure 2:**
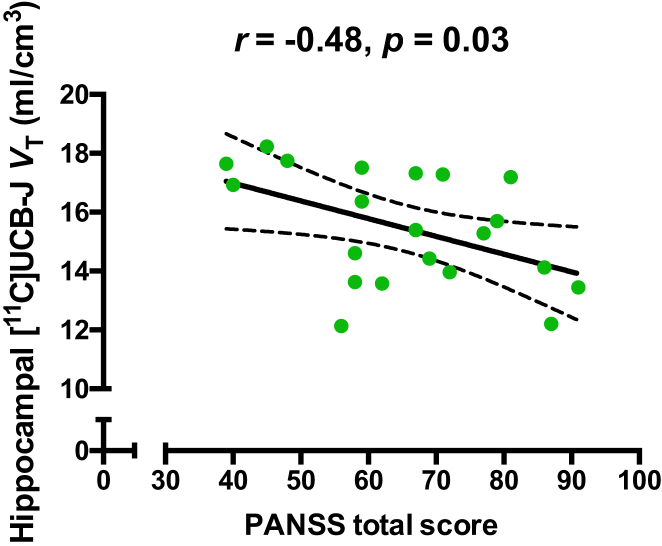
Significant negative association between hippocampal [^11^C]UCB-J volume of distribution (*V*_T_) and Positive and Negative Syndrome Scale (PANSS) total score.

In the SCZ group, mean (SEM) PANSS scores were as follows: total score, 65.29 (3.29); positive, 17.05 (1.24); negative, 17.81 (0.95); and general, 30.43 (1.81). In the SCZ group, there was a significant negative relationship between hippocampal [^11^C]UCB-J *V*_T_ and the PANSS total score (*r* = −0.48, *p* = .03) (Figure 2; Table S2) and a nonsignificant trend toward a negative relationship for *V*_T_ with positive (*r* = −0.42, *p* = .06) and general (*r* = −0.42, *p* = .06) but not negative PANSS subscale scores (Figures S1–S3). There were no significant relationships between FC or ACC [^11^C]UCB-J *V*_T_ and PANSS total scores (Figures S4, S5; Table S2).

### Exploratory Analysis of [^11^C]UCB-J *V*_T_/*f*_p_ in Primary ROIs

Given the finding of numerically greater *f*_p_ in the SCZ group than in the HV group, we calculated *V*_T_/*f*_p_ as an alternative outcome measure to correct for differences in *f*_p_. There was a significant effect of ROI (*F*_2,67_ = 722.07, *p* < .0005) and of the group-by-ROI interaction (*F*_2,67_ = 3.83, *p* = .03) but not of group (*F*_1,40_ = 3.91, *p* = .06) on [^11^C]UCB-J *V*_T_/*f*_p_. Post hoc analyses revealed that *V*_T_/*f*_p_ was significantly lower in the SCZ group in the ACC [mean (SEM) HV = 91.71 (1.93), SCZ = 83.79 (2.70), *t*_40_ = 2.38, *p* = .02, *d* = 0.7], although this finding did not survive FDR adjustment for multiple comparisons, and *V*_T_/*f*_p_ was not significantly altered in the FC or hippocampus (Table S3).

### Exploratory Analysis of [^11^C]UCB-J *V*_T_ in Other Regions

There was a significant effect of ROI (*F*_5,100_ = 135.9, *p* < .0001) but not group (*F*_1,20_ = 0.04, *p* = .85), even with age as a covariate (*F*_1,39_ = 0.86, *p* = .34), on [^11^C]UCB-J *V*_T_ in the exploratory ROIs.

### Whole-Brain Analyses

A voxelwise whole-brain analysis showed that there were no significant differences between patients and control participants in [^11^C]UCB-J *V*_T_ (familywise error–corrected *p* < .05), and this remained the case even when we used a liberal threshold (uncorrected *p* < .001).

### [^11^C]UCB-J *V*_T_ in the CS

Data were not normally distributed in the SCZ group. There was no significant group difference in mean (SEM) [^11^C]UCB-J *V*_T_ in the CS [HV = 5.54 (0.13); SCZ = 6.34 (0.37); Kolmogorov-Smirnov *z* = 0.93, *p* = .36] (Table S1; Figure S6).

### [^11^C]UCB-J DVR Across Groups

**Figure 3:**
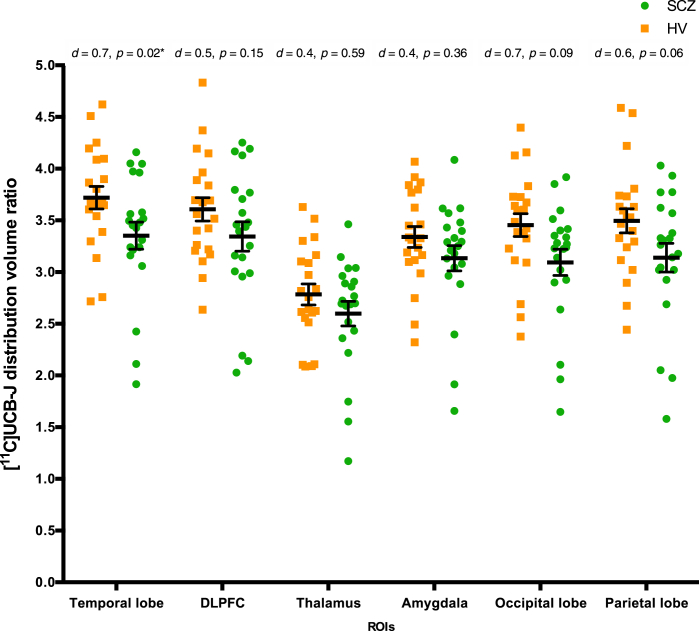
Mean [^11^C]UCB-J distribution volume ratio in the occipital, parietal, and temporal lobes; the dorsolateral prefrontal cortex (DLPFC); and the thalamus and amygdala, by group. Orange squares indicate the healthy volunteer (HV) group (*n* = 21); green dots indicate the schizophrenia (SCZ) group (*n* = 21). [^11^C]UCB-J distribution volume ratio was not significantly altered in any region of interest (ROI) following adjustment for multiple comparisons. ∗ indicates *p* value that did not survive false discovery rate adjustment. Error bars indicate standard error of the mean.

The mean [^11^C]UCB-J DVR was significantly lower in the SCZ group than in the HV group in the temporal lobe (Kolmogorov-Smirnov *z* = 1.54, *p* = .02, Cohen’s *d* = 0.7) (Figure 3), although this finding did not survive FDR adjustment for multiple comparisons. DVR was not significantly different between groups in any other regions, although values were numerically lower in the SCZ group than in the HV group (Supplemental Results; Tables S4 and S5; Figure 3; Figure S7).

### Relationship Between [^11^C]UCB-J DVR and Symptom Severity

In the SCZ group, there were no significant relationships between [^11^C]UCB-J DVR in the FC, ACC, or hippocampus and PANSS scores (Table S6). Given the exploratory finding of lower [^11^C]UCB-J DVR in the temporal lobe in the SCZ group, we also tested relationships between [^11^C]UCB-J DVR and PANSS scores in this region but found no significant relationships (Table S6).

### Relationship Between [^11^C]UCB-J and Duration of Antipsychotic-Free Interval

There were no significant relationships between the duration of the interval since the most recent antipsychotic treatment and [^11^C]UCB-J *V*_T_ or DVR in any ROI (Supplemental Results).

## Discussion

The main finding of this study is that [^11^C]UCB-J *V*_T_, an in vivo marker of synaptic vesicle glycoprotein 2A (SV2A) levels and a proxy marker of presynaptic terminal density, is not significantly different between early-course SCZ and HV groups across the brain. However, hippocampal [^11^C]UCB-J *V*_T_ is negatively associated with total PANSS score.

Our exploratory analyses using alternative outcome measures found evidence for lower DVR in the temporal lobe and lower *V*_T_/*f*_p_ in the ACC in patients with early-course SCZ than in HVs, although neither finding survived correction for multiple comparisons. We also found trends toward lower DVR in the FC, ACC, and occipital and parietal lobes in patients with early-course SCZ than in HVs. [^11^C]UCB-J DVR data show lower variability than *V*_T_ data (7). The increased sensitivity of the DVR approach to detect group differences could explain why there were trends for group differences in DVR but not *V*_T_. Another consideration is that DVR adjusts for nonspecific binding, while *V*_T_ indexes total tracer distribution in the tissue. Thus, trends toward lower DVR and *V*_T_/*f*_p_ values in patients could reflect lower SV2A levels that are masked in the *V*_T_ data by nonspecific binding, subtle *f*_p_ differences, and/or the lower sensitivity of the *V*_T_ approach compared with the DVR approach. Nevertheless, our findings differ from the findings of studies conducted with chronic, treated samples, in which *V*_T_ and DVR were both lower in SCZ, with large effect sizes (7), indicating that if there are SV2A deficits in early-course, untreated patients compared with control participants, they are likely to be less marked than in chronic, treated patients.

The patients in the previous studies had a mean illness duration of 17.4 years (7) and 17.3 years (8) and were mostly being treated with antipsychotic medication. In contrast, the mean illness duration of patients in the current study was 2.7 years, and none of our patients were taking antipsychotic medication. Thus, differences between the findings from the current study and earlier findings may be due to illness duration and/or antipsychotic exposure effects on SV2A levels (7,8). However, a rat study found no effect of antipsychotic treatment on SV2A markers (7), and the previous [^11^C]UCB-J studies in chronic SCZ found no relationship between antipsychotic exposure and [^11^C]UCB-J distribution volume in patients (7,8). Moreover, in this study, we found no association between drug-free interval duration and SV2A levels. Taken together, the evidence indicates that the difference between our current findings and earlier SV2A findings in SCZ is unlikely to be due to antipsychotic treatment effects. Instead, the differences could be due to differences in illness chronicity.

Another consideration is that patient ages differed between studies. In the earlier [^11^C]UCB-J studies in SCZ (7,8), the mean patient age was 41.5 and 40.5 years, respectively, whereas it was 26.5 years in the current study. Postmortem evaluations have indicated that dendritic spine density declines throughout the third decade of human life before stabilizing at around 30 years of age (21). It is possible that any illness effects on developmental synaptic pruning manifest only as small differences in SV2A levels during the third decade of life, in contrast to those identified during the fifth decade. However, the current study was only adequately powered to capture large effects. Moreover, age did not differ significantly between our groups, and findings remained essentially the same when age was added as a covariate to the analyses.

In the current study, mean PANSS scores were higher than in our previous study conducted with patients with chronic SCZ (7). Thus, the early-course group reported on here showed greater symptom severity than the chronic SCZ group that we reported on previously. The significant negative relationship between PANSS total scores and [^11^C]UCB-J *V*_T_ in the hippocampus confirmed our hypothesis that SV2A levels are inversely associated with symptom severity, thereby extending preclinical and clinical evidence implicating hippocampal dysfunction in SCZ (14,22, 23, 24, 25), but contrasting with SV2A findings in chronic patients (7). Speculatively, these findings could suggest that SV2A levels are related to the disease process in early-course but not in chronic SCZ. Alternatively, the difference could be due to antipsychotic treatment in the chronic patients reducing symptom severity, hence the strength of the relationship with SV2A levels in the chronic SCZ study. Our finding of a link between SV2A levels and overall symptom severity in the hippocampus was not corrected for multiple comparisons, and we did not find this relationship in the FC or ACC or an association between [^11^C]UCB-J DVR and PANSS scores. Thus, additional studies are needed to test the link between SV2A measures and symptoms further.

### Strengths and Limitations

Strengths of the current study include the fact that it is the first study to our knowledge to investigate SV2A levels early in the course of SCZ in untreated patients. While none of the patients included in this study were currently taking antipsychotic medication, most (*n* = 19) had previously done so. Thus, it is possible that prior antipsychotic treatment influenced our findings. This could explain why we found no significant differences between unmedicated patients and control participants in SV2A levels, whereas groups of medicated (7) and largely medicated patients (*n* = 10 of 13) (8) have shown large deficits in SV2A levels in diverse brain regions. However, the antipsychotics that patients had previously taken are not known to directly bind to SV2A (26). Moreover, previous studies have found no link between prior antipsychotic exposure and SV2A binding in the rat or human brain (7,8), and in this study, patients had a mean wash-out from antipsychotic treatment of 183 (minimum 41) days. Thus, it is unlikely that prior antipsychotic treatment explains our findings. Nevertheless, additional work is needed to test for the effects of antipsychotic exposure on SV2A levels.

It should be considered that the PET imaging has a transaxial spatial resolution of 4.6 mm and so is insensitive to alterations in regions smaller than this. Postmortem studies have identified lamina specificity in dendritic spine density alterations in SCZ compared with control subjects (27), particularly in cortical layer 3 (28, 29, 30). Thus, it remains possible that there are substructural group differences in SV2A levels that are obscured by PET resolution limits.

We used the CS, a white matter region largely devoid of SV2A, as a reference region to adjust for nonspecific binding when estimating [^11^C]UCB-J DVR (18). [^11^C]UCB-J DVR may reflect the SV2A-specific signal more closely than it reflects *V*_T_ (31). However, white matter may serve suboptimally as a reference region given its distinct tissue composition from gray matter (31). Moreover, levetiracetam, an SV2A-selective drug, displaces a small amount of [^11^C]UCB-J in the CS, indicating low specific binding levels in this region (18). Group differences in CS-specific binding could have biased our findings, although we found no significant group differences in CS [^11^C]UCB-J *V*_T_. Thus, our DVR findings suggest a trend toward lower levels of SV2A-specific binding in patients with early-course, untreated SCZ than in control participants. However, our finding of lower temporal lobe DVR did not survive correction for multiple comparisons.

Finally, our study was powered to detect group differences of the magnitude previously detected in chronic SCZ, but a type II error is possible, and we were underpowered to detect more subtle differences. Notwithstanding this, inspection of the *V*_T_ data (Figure 1) does not indicate a trend toward more subtle differences. Nevertheless, additional studies with early-course patients are warranted to confirm our findings.

### Interpretation and Implications for the Synaptic Hypothesis of SCZ

[^11^C]UCB-J shows high specific binding to SV2A (32) and regional uptake consistent with prior anatomical knowledge regarding synaptic distribution (18). In gray matter, [^11^C]UCB-J shows displacement by levetiracetam, a drug specific to SV2A, indicating specific binding to SV2A (18). It also shows good test-retest reproducibility (18,19), indicating that [^11^C]UCB-J is a reliable SV2A-binding PET radioligand. SV2A is found ubiquitously in synaptic terminals throughout gray matter (33). Moreover, in the baboon brain, regional levels of SV2A are strongly positively correlated with those of synaptophysin (*r* > 0.95), which is often regarded as a gold standard ex vivo measure of synaptic density (18). It is possible that synaptic terminal density is lower in the absence of altered SV2A levels in early-course SCZ. However, this would require there to be greater SV2A levels per synaptic terminal in our patients, and when taken together with the evidence of lower SV2A levels in chronic patients, it would require this process to reverse at some point or for there to be very marked synaptic terminal density loss, which is not consistent with postmortem findings (3). We are unaware of any mechanism that could account for this. An alternative, more parsimonious explanation is that there are no, or subtle, synaptic terminal density deficits early in the course of SCZ and that these develop during the course of the illness, which would explain SV2A and postmortem synaptic density findings in patients with chronic illness (3,7,9,28), and be consistent with previous evidence for brain volume loss during the course of SCZ (34, 35, 36).

One mechanism that could account for synaptic changes in SCZ involves microglia (37,38). Microglia play a key role in synaptic pruning (39, 40, 41). Several lines of evidence indicate that this process may be abnormal in SCZ [reviewed in (42)], including findings of greater microglia-mediated synaptic engulfment in patient-derived neuronal preparations (43). Notwithstanding the caveats mentioned above, our current findings are not consistent with versions of the synaptic hypothesis that propose early developmental failure to form synapses and/or excess developmental synaptic pruning before illness onset (44,45). However, given that SV2A is a presynaptic marker, it remains unknown whether there are changes in postsynaptic density markers at illness onset in SCZ. Postsynaptic markers such as dendritic spine density and PSD-95 levels are significantly lower in SCZ in a meta-analysis of postmortem studies (28) and in numerous animal models of genetic (46,47) and environmental (48, 49, 50, 51, 52) risk factors for subjects with SCZ when compared to control subjects. The development of in vivo imaging probes specific to postsynaptic markers would be invaluable in assessing whether postsynaptic density changes emerge in patients during the course of SCZ. Moreover, the proposal that synaptic density changes occur during the course of illness remains speculative at this time, and it is important to consider alternative explanations. In particular, cohort effects, such as lower synaptic levels being specific to a subgroup of patients who are more likely to develop chronic illness, could account for differences between our current findings and previous findings with chronic patients. The generalizability of our sample to other settings should be considered. However, epidemiological and clinical trial data in first-episode samples have shown that ∼30% to 70% of patients discontinue antipsychotic treatment (53,54), indicating that our sample is not unusual in this respect. Longitudinal studies are needed to determine whether there are progressive SV2A reductions in SCZ over time.

### Conclusions

Levels of [^11^C]UCB-J binding, a synaptic terminal marker, are not significantly different in patients with early-course SCZ compared with healthy volunteers, but we found preliminary evidence in exploratory analyses that there may be lower levels in the temporal lobe and some other brain regions and that greater symptom severity is associated with lower hippocampal synaptic marker levels. These findings are not consistent with our hypothesis that marked synaptic deficits are present early in the course of SCZ and indicate that the lower SV2A levels identified in patients with chronic SCZ may develop after illness onset, although longitudinal studies are warranted to confirm this.

## Supplementary Material

Supplemental Material

Key Resources Table
