# Supplementary material for: Synaptic Terminal Density Early in the Course of Schizophrenia: An In Vivo UCB-J Positron Emission Tomographic Imaging Study of SV2A
Source: Biol Psychiatry. 2024 Apr 1;95(7):639–46. doi: 10.1016/j.biopsych.2023.05.022 (PMC10923626; doi:10.1016/j.biopsych.2023.05.022)
Supplement: Supplemental Material [file mmc1.pdf]

SUPPLEMENTARY INFORMATION

Synaptic Terminal Density Early in the Course of Schizophrenia: An In Vivo UCB-J Positron Emission Tomographic Imaging Study of Synaptic Vesicle Glycoprotein 2A

Onwordi *et al.*

Supplementary methods

Exclusion criteria for all volunteers were: history of head trauma resulting in a loss of consciousness; drug or alcohol dependence (except for nicotine dependence); neurological disorder; significant medical disorder; taking drugs known to interact with SV2A (e.g. levetiracetam, brivaracetam, loratadine or quinine (1)); or contraindications to imaging. Age, gender, and smoking status were recorded.

Clinical assessments

Psychiatric symptoms were assessed by a research clinician using the Structured Clinical Interview for DSM-5 to confirm the diagnosis and assess for psychiatric co-morbidities, and the PANSS to evaluate symptom severity (2). Illness duration was determined as the time from first psychotic symptoms. Healthy volunteers were screened using the Structured Clinical Interview for DSM-5 to exclude any psychiatric illness and to exclude any family history of psychosis.

MR imaging

All subjects underwent structural magnetic resonance imaging (MRI) to facilitate the anatomical delineation of regions of interest (ROIs). For all patients and 19 healthy volunteers, T1-weighted three-dimension magnetisation-prepared rapid acquisition gradient echo (MPRAGE) images were acquired on a Siemens Magnetom Prisma 3T scanner (Siemens, Erlangen, Germany) with the following parameters: repetition time = 2300.0ms, echo time = 2.28ms, flip angle = 9°, field of view = 256 × 256mm, 176 sagittal slices of 1-mm thickness, distance factor = 50%, voxel size = 1.0 × 1.0 × 1.0mm. For two healthy volunteers, T1-weighted three-dimension MPRAGE images were acquired on a Siemens 3T

Trio clinical MRI scanner (Siemens Healthineers, Erlangen, Germany) with the following parameters: repetition time = 2300.0ms, echo time = 2.98ms, flip angle = 9°, field of view = 256 × 256mm, 160 sagittal slices of 1-mm thickness, distance factor = 50%, voxel size = 1.0 × 1.0 × 1.0mm.

PET imaging

Subjects underwent [^{11}C]UCB-J scanning between March 2018 and April 2022: SCZ subjects between April 2019 and February 2022; HV subjects between March 2018 and April 2022. There were no changes in procedures, software or hardware during the study.

Arterial blood sampling

Radial arterial blood samples were collected throughout the PET scan to measure the arterial input function as detailed elsewhere (3). Briefly, a continuous automatic blood sampling system was used to measure whole blood activity for the first fifteen minutes (Allogg AB, Mariefred, Sweden). Discrete samples were taken 10, 15, 20, 25, 30, 40, 50, 60, 70, 80 and 90 minutes after tracer injection. A Perkin Elmer 1470 10-well gamma counter was used to measure total blood and plasma radioactivity concentrations. High-performance liquid chromatography was used to evaluate the plasma radioactivity fraction constituted by unchanged parent radioligand from discrete blood samples. Ultrafiltration in triplicate was used to measure the [^{11}C]UCB-J plasma free fraction from an arterial blood sample taken before tracer injection.

Voxelwise whole-brain analysis

Exploratory voxelwise, whole-brain, analyses were conducted using SPM12 to determine whether there were alterations in [^{11}C]UCB-J V_T not detected by the region-of-interest analysis. To do this, we first produced individual [^{11}C]UCB-J V_T voxelwise parametric maps

of the whole brain using the basis function implementation of the one-tissue compartment model. These individual images were then spatially normalised to the MNI152 template using nonlinear registration, enabling the production of mean parametric images for each group. An independent samples t-test was used to investigate if there were voxelwise differences in whole-brain grey matter [^{11}C]UCB-J V_T between patients and healthy volunteers. The extent threshold was set to k=20 voxels and peak-level family-wise error corrected thresholds (p<0.05) were used in the primary analysis. To explore the possibility of subtle differences, we repeated this analysis using a liberal threshold (uncorrected p<0.001).

Supplementary results

[^{11}C]UCB-J DVR across groups

Data were not normally distributed in the SCZ group in the anterior cingulate cortex (ACC, Shapiro-Wilk W = 0.88, p = 0.01) or the hippocampus (Shapiro-Wilk W = 0.86, p = 0.006), and so non-parametric analyses were used for these ROIs. Mean (SEM) [^{11}C]UCB-J DVR was not significantly altered between groups in the frontal cortex (FC, SCZ = 3.28 [0.14]; HV = 3.56 [0.11]; t = 1.58, df = 40.0, p = 0.12, Cohen's d = 0.5), ACC (SCZ = 3.66 [0.14]; HV = 4.05 [0.11]; Kolmogorov-Smirnov Z = 1.23, p = 0.09, Cohen's d = 0.7) or hippocampus (SCZ = 2.54 [0.11]; HV = 2.73 [0.08]; Kolmogorov-Smirnov Z = 0.62, p = 0.84, Cohen's d = 0.4; Supplementary Figure 7, Supplementary Table 4).

[^{11}C]UCB-J DVR across groups in exploratory ROIs

Data were not normally distributed in the SCZ group in the temporal lobe (Shapiro-Wilk W = 0.89, p = 0.02), thalamus (Shapiro-Wilk W = 0.89, p = 0.03), amygdala (Shapiro-Wilk W = 0.87, p = 0.01), or occipital lobe (Shapiro-Wilk W = 0.88, p = 0.01), and so non-parametric

analyses were used for these ROIs. Mean [SEM] [^{11}C]UCB-J DVR was lower in the SCZ relative to the HV group in the temporal lobe (SCZ = 3.35 [0.13]; HV = 3.72 [0.11]; Kolmogorov-Smirnov $Z = 1.54$, $p = 0.02$, Cohen's $d = 0.7$), although this finding did not survive FDR adjustment for multiple comparisons. Mean [SEM] [^{11}C]UCB-J DVR was not significantly altered in the SCZ relative to the HV group in any other ROI after FDR adjustment for multiple comparisons (Figure 3, Supplementary Table 5).

Relationship between [^{11}C]UCB-J V_T and duration of the drug-free interval

In the 19 patients in SCZ group who had previously taken antipsychotic treatment, there were no significant relationships between the duration of the interval between last antipsychotic treatment and [^{11}C]UCB-J V_T in the FC (Spearman's $\rho = -0.29$, $p = 0.41$), ACC ($\rho = -0.11$, $p = 0.64$), hippocampus ($\rho = -0.09$, $p = 0.73$), temporal lobe ($\rho = -0.11$, $p = 0.66$), DLPFC ($\rho = -0.18$, $p = 0.46$), thalamus ($\rho = -0.18$, $p = 0.46$), amygdala ($\rho = -0.20$, $p = 0.42$), occipital lobe ($\rho = -0.14$, $p = 0.56$), or parietal lobe ($\rho = -0.17$, $p = 0.50$).

Relationship between [^{11}C]UCB-J DVR and duration of the drug-free interval

In the 19 patients in SCZ group who had previously taken antipsychotic treatment, there were no significant relationships between the duration of the interval between last antipsychotic treatment and [^{11}C]UCB-J DVR: frontal cortex (Spearman's $\rho = 0.14$, $p = 0.57$), ACC ($\rho = 0.20$, $p = 0.39$), hippocampus ($\rho = 0.15$, $p = 0.53$), temporal lobe ($\rho = 0.33$, $p = 0.17$), DLPFC ($\rho = 0.20$, $p = 0.42$), thalamus ($\rho = 0.03$, $p = 0.92$), amygdala ($\rho = 0.22$, $p = 0.38$), occipital lobe ($\rho = 0.32$, $p = 0.19$), parietal lobe ($\rho = 0.19$, $p = 0.45$).

Supplementary Table 1 Mean and standard error of mean (SEM) [^{11}C]UCB-J V_T in healthy volunteer (HV) and schizophrenia (SCZ) groups. Test statistic = t, except where asterisk indicates Kolmogorov-Smirnov test used due to data not being normally distributed (where test statistic = Z).

	[^{11}C]UCB-J V_T ml/cm^3				*Test statistic*	df	*p*	Cohen's *d*
	HV		SCZ					
	Mean	SEM	Mean	SEM				
Frontal cortex	19.63	0.65	20.01	0.65	0.41	40	0.69	0.1
ACC	22.37	0.70	22.37	0.62	0.004	40	1.00	0.0
Hippocampus	15.04	0.50	15.46	0.42	0.65	40	0.52	0.2
Centrum semiovale*	5.54	0.13	6.34	0.37	0.93	-	0.36	0.6

Supplementary Table 2 Associations between PANSS scores (total and positive, negative and general subscale scores) and [^{11}C]UCB-J volume of distribution (V_T) in the hippocampus, frontal cortex and anterior cingulate cortex, explored in the schizophrenia group (n = 21). There was a significant negative association between hippocampal [^{11}C]UCB-J V_T and PANSS total score in the SCZ group. No other significant associations were detected. All correlation coefficients reported are Pearson product-moment correlation coefficients.

		**PANSS total**		**PANSS positive**		**PANSS negative**		**PANSS general**	
		Correlation coefficient	*P* value	Correlation coefficient	*P* value	Correlation coefficient	*P* value	Correlation coefficient	*P* value
**[^{11}C]UCB-J V_T**	Hippocampus	-0.48	0.03	-0.42	0.06	-0.31	0.18	-0.42	0.06
	Frontal cortex	-0.17	0.45	-0.18	0.44	-0.08	0.73	-0.15	0.51
	Anterior cingulate cortex	-0.22	0.33	-0.24	0.30	-0.14	0.55	-0.17	0.46

Supplementary Table 3 Mean and standard error of mean (SEM) [^{11}C]UCB-J V_T/f_p in healthy volunteer (HV) and schizophrenia (SCZ) groups. Test statistic = t, except where asterisk indicates Kolmogorov-Smirnov test used due to data not being normally distributed (where test statistic = Z). Obelisk (†) indicates p value not surviving FDR adjustment for multiple comparisons.

	[^{11}C]UCB-J V_T/f_p ml/cm^3				*Test statistic*	df	*p*	Cohen's *d*
	HV		SCZ					
	Mean	SEM	Mean	SEM				
Frontal cortex	80.49	1.90	74.95	2.67	1.69	40	0.10	0.5
ACC	91.71	1.93	83.79	2.70	2.38	40	0.02†	0.7
Hippocampus	61.72	1.62	57.92	1.80	1.57	40	0.12	0.6

Supplementary Table 4 Mean and standard error of mean (SEM) [^{11}C]UCB-J distribution volume ratio (DVR) in healthy volunteer (HV) and schizophrenia (SCZ) groups in frontal cortex, anterior cingulate cortex (ACC) and hippocampus. Test statistic = t, except where asterisk indicates Kolmogorov-Smirnov test used due to data not being normally distributed (where test statistic = Z).

	[^{11}C]UCB-J DVR				*Test statistic*	df	*p*	Cohen's *d*
	HV		SCZ					
	Mean	SEM	Mean	SEM				
Frontal cortex	3.56	0.11	3.28	0.14	1.58	40	0.12	0.5
ACC*	4.05	0.11	3.66	0.14	1.23	-	0.09	0.7
Hippocampus*	2.73	0.08	2.54	0.11	0.62	-	0.84	0.4

Supplementary Table 5 Mean and standard error of mean (SEM) [^{11}C]UCB-J DVR in healthy volunteer (HV) and schizophrenia (SCZ) groups in exploratory regions of interest. Test statistic = t, except where asterisk (*) indicates Kolmogorov-Smirnov test used due to data not being normally distributed (where test statistic = Z). Obelisk (†) indicates p value not surviving FDR adjustment for multiple comparisons.

	[^{11}C]UCB-J DVR				*Test statistic*	df	*p*	Cohen's *d*
	HV		SCZ					
	Mean	SEM	Mean	SEM				
Temporal lobe*	3.72	0.11	3.35	0.13	1.54	-	0.02†	0.7
DLPFC	3.61	0.11	3.34	0.14	1.45	40.00	0.15	0.5
Thalamus*	2.78	0.10	2.60	0.12	0.77	-	0.59	0.4
Amygdala*	3.34	0.10	3.13	0.12	0.93	-	0.36	0.4
Occipital lobe*	3.45	0.11	3.09	0.13	1.23	-	0.09	0.7
Parietal lobe	3.50	0.12	3.14	0.14	1.97	40.00	0.06	0.6

Supplementary Table 6 Associations between PANSS scores (total and positive, negative and general subscale scores) and [11C]UCB-J distribution volume ratio (DVR) in the hippocampus, frontal cortex, anterior cingulate cortex and temporal lobe, explored in the schizophrenia group (n = 21). No significant associations between [11C]UCB-J DVR and PANSS scores were detected. All correlation coefficients reported are Pearson product-moment correlation coefficients, except where asterisk indicates Spearman's correlation coefficient used due to data not being normally distributed.

		**PANSS total**		**PANSS positive**		**PANSS negative**		**PANSS general**	
		Correlation coefficient	*P* value	Correlation coefficient	*P* value	Correlation coefficient	*P* value	Correlation coefficient	*P* value
**[11C]UCB-J DVR**	Hippocampus	-0.26	0.26	-0.12	0.62	-0.12	0.61	-0.33	0.15
	Frontal cortex	-0.13	0.58	-0.01	0.96	-0.01	0.97	-0.22	0.34
	Anterior cingulate cortex	-0.15	0.58	-0.03	0.89	-0.04	0.85	-0.22	0.33
	Temporal lobe*	0.06	0.80	0.09	0.70	0.09	0.67	-0.05	0.83

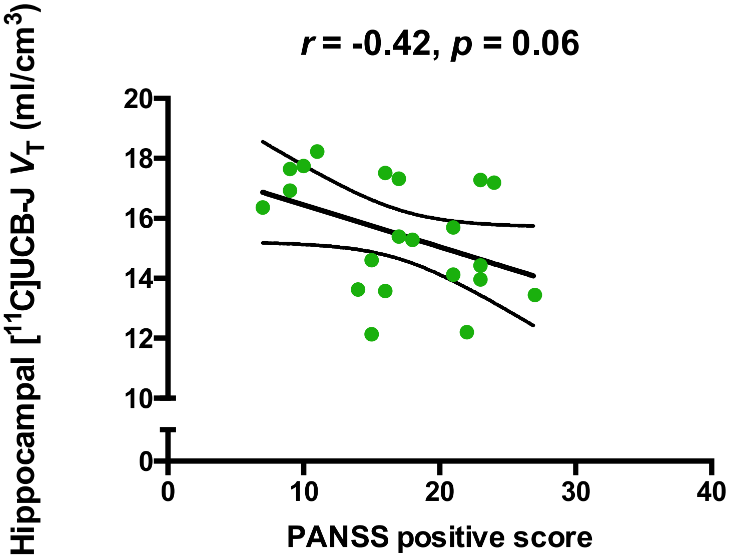

Supplementary Figure 1 No significant association between hippocampal [^{11}C]UCB-J volume of distribution (V_T) and PANSS positive score.

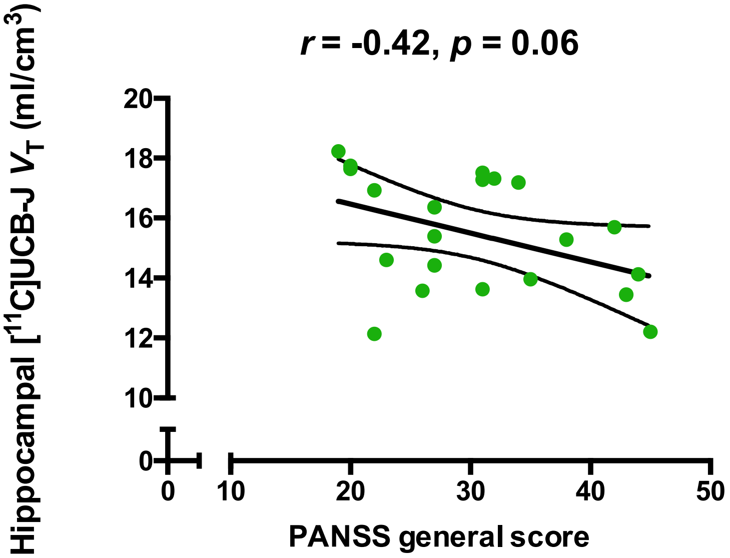

Supplementary Figure 2 No significant association between hippocampal [^{11}C]UCB-J volume of distribution (V_T) and PANSS general score.

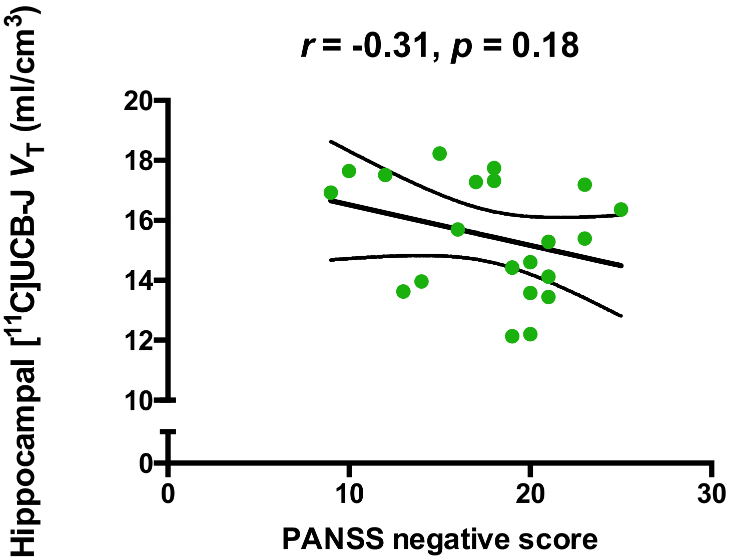

Supplementary Figure 3 No significant association between hippocampal [^{11}C]UCB-J volume of distribution (V_T) and PANSS negative score.

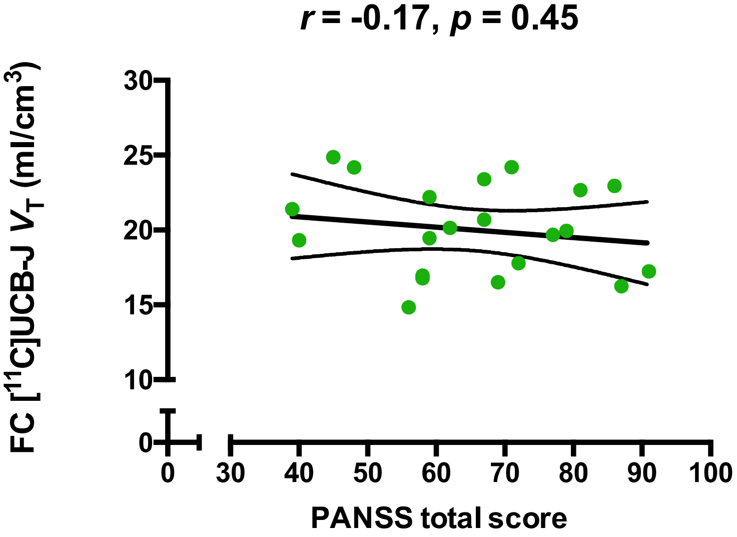

Supplementary Figure 4 No significant association between frontal cortical (FC) [^{11}C]UCB-J volume of distribution (V_T) and PANSS total score.

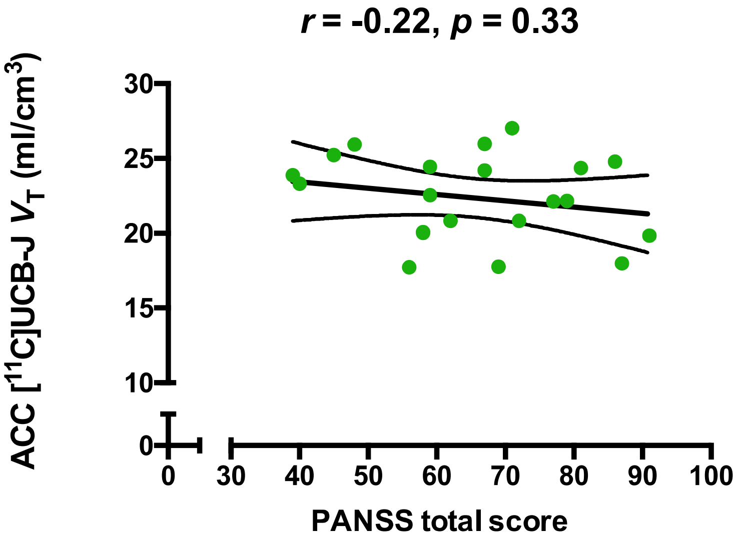

Supplementary Figure 5 No significant association between anterior cingulate cortical (ACC) [^{11}C]UCB-J volume of distribution (V_T) and PANSS total score.

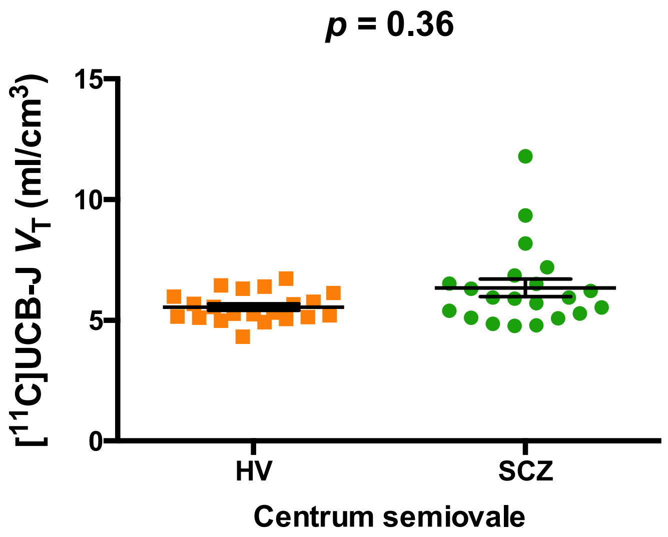

Supplementary Figure 6 [^{11}C]UCB-J distribution volume (V_T) in the centrum semiovale by group. Orange squares indicate the healthy volunteer group (n = 21); green dots indicate the schizophrenia group (n = 21). [^{11}C]UCB-J V_T was significantly not significantly altered in schizophrenia patients compared to controls in the centrum semiovale. The horizontal bar is the mean, error bars indicate standard error of the mean.

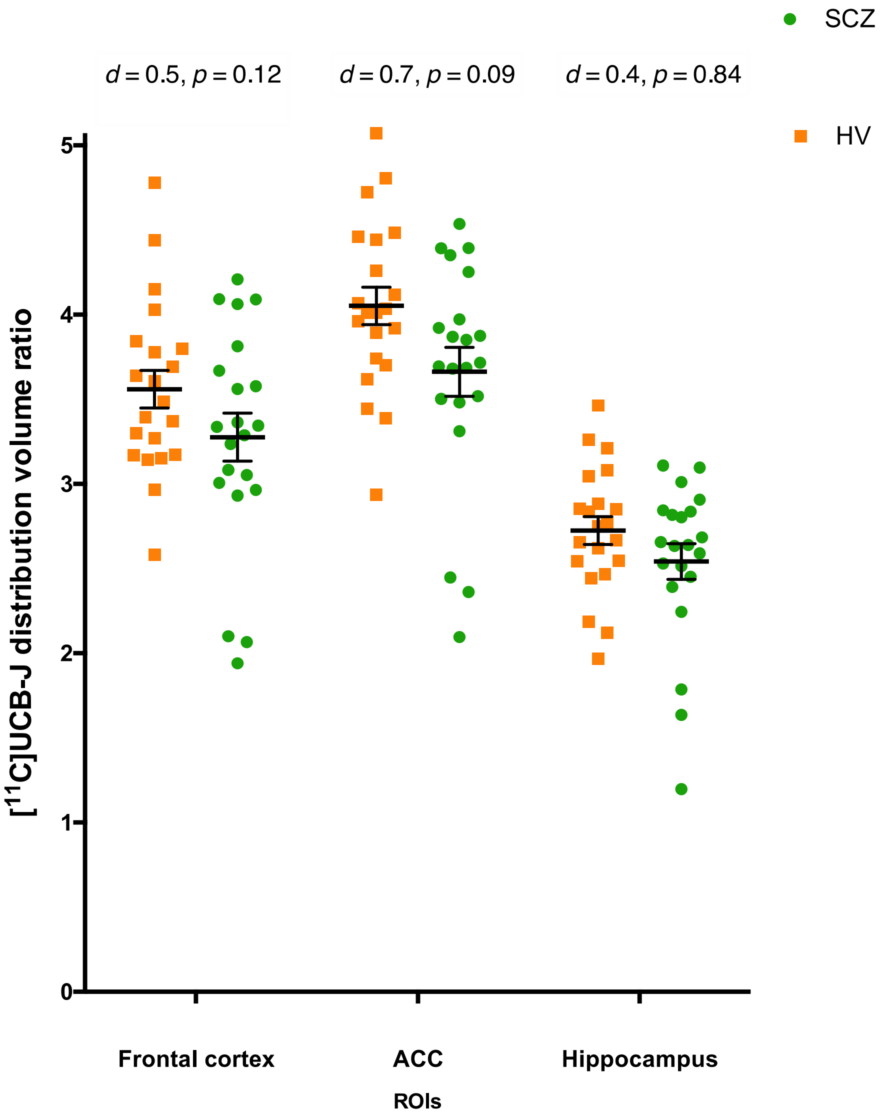

Supplementary Figure 7 Exploratory analysis investigating [^{11}C]UCB-J distribution volume ratio (DVR) in the frontal cortex, anterior cingulate cortex and hippocampus by group. Orange squares indicate the healthy volunteer group (n = 21); green dots indicate the schizophrenia group (n = 21). [^{11}C]UCB-J DVR was not significantly altered in any region of interest in schizophrenia patients compared to healthy volunteers. Horizontal bar indicates the mean, error bars indicate standard error of the mean.

References

1. Danish A, Namasivayam V, Schiedel AC, Muller CE. Interaction of Approved Drugs with Synaptic Vesicle Protein 2A. Arch Pharm (Weinheim). 2017;350(3-4).

2. Kay SR, Fiszbein A, Opler LA. The Positive and Negative Syndrome Scale (PANSS) for Schizophrenia. Schizophrenia Bulletin. 1987;13(2):261-76.

3. Mansur A, Rabiner EA, Comley RA, Lewis Y, Middleton LT, Huiban M, et al. Characterization of 3 PET tracers for Quantification of Mitochondrial and Synaptic function in Healthy Human Brain: (18)F-BCPP-EF, (11)C-SA-4503, (11)C-UCB-J. J Nucl Med. 2019.
